# Pathways to and Consequences of Surrogate Decision-Making for Older Adults

**DOI:** 10.1093/geroni/igaa057.2466

**Published:** 2020-12-16

**Authors:** Pamela Teaster, Cory Bolkan, Shawn Meyers

## Abstract

With a burgeoning aging population, there is a growing need for surrogate decision makers, yet oversight of and guidance for them remains inadequate. People needing surrogate decision makers are an especially vulnerable population because they rely on others for care and/or are unable to advocate for themselves. Their vulnerability leaves them susceptible to elder abuse (e.g., physical, sexual, psychological abuse; active and passive neglect; financial exploitation), which affects approximately five million older Americans each year. Personal, financial, and societal impacts can be devastating and are estimated to cost billions annually. The issue of abuse, neglect and exploitation by surrogates has been highly visible nationally, evidence indicates that some surrogate decision makers perpetrate abuse. One purpose of this symposium is to discuss ways in which surrogates do and do not make decisions for older adults. Ramsey-Klawsnik and Burnett present data at the systemic level to illustrate how self-neglect sequelae can result in placement under surrogate decision-making authority of either well-intended or opportunistic others. Bolkan, Teaster, Ramsey-Klawsnik, and Gerow present findings from a six-state study on surrogate decision maker victims and perpetrators who were substantiated in Adult Protective Services cases. Zhao, Katz, and Teaster show, using a survey of M-Turk participants, how a general population makes and is comfortable with surrogate decisions. Discussant Shawn Meyers will pull together the findings by exploring their translation to judicial best practices for making determinations regarding surrogate decision makers and the effects of their decisions on the surrogate as well as collaterals.

